# Obstetrics and Gynecology

**Published:** 1896-06

**Authors:** 


### SELECTIONS AND ABSTRACTS.

### OBSTETRICS AND GYNECOLOGY.

EDITED BY DR. VIRGIL O. HARDON.

### PUS IN THE PELVIS.

Dr. Thomas A. Ashby, in a paper read before the Gynecological and Obstetrical Society of Baltimore, and published in *The American Journal of Obstetrics,* considers the dangers of delay in dealing with pus in the pelvis. He writes that whether the patient be in imminent danger or not, prompt interference in dealing with pus, especially pus in the pelvis, cannot be too soon acted upon. He says:

"The pus sac is a further source of danger from the fact that it invites repeated attacks of intrapelvic inflammation, which in turn lead to the formation of adhesions between the sac wall and surrounding tissues. The removal of the sac is in this way made more difficult and dangerous, whilst early removals are attended with less danger to life and with a surer restoration to health. All who have removed these old adherent sac walls can appreciate the difficulties and dangers of such procedures and the sequelæ which not infrequently follow after recovery from abdominal section. How often is it that the abdomen has to be reopened a second or third time for the drainage of newly-formed pus accumulations from an old infected area left behind! For, remove these old pus sacs with all the care possible, we cannot always feel sure that every particle of the infected sac wall has been removed.

"Chronic pus sacs in the pelvis involve risk to life in their remote influences. The local condition may create comparatively little local pain or disturbance. The patient may enjoy such an exemption from pelvic disturbances as to mislead her attending physician in his diagnosis of her true condition. Pus formation and pus absorption may go on in such a passive way that the symptoms of the case are referred to such general bodily conditions as anemia, chlorosis, malarial fever, and, as in one case I once operated on, which for weeks was treated for typhoid fever, when neither death nor recovery followed, a new doctor and a new diagnosis discovered a large pelvic pus accumulation. The patient recovered promptly after the removal of the pus sac. The remote effects of pus are almost as pernicious as its immediate effects. The difference in results is found in the rapidity and violence with which symptoms follow in the latter conditions, often overwhelming the patient with the explosive force of the inflammatory or septic action, whilst in the former cases chronic blood changes and nutritive disturbances of heart, kidneys, and other organs are the most usual evidences of pus absorption."—*Am. Med. Review.*

---

### THE TREATMENT OF THE STUMP OF THE UMBILICAL CORD.

In his new book on the "Therapeutics of Infancy and Childhood," Dr. A. Jacobi says:

"In wrapping up the end of the cord no oil must be used. Warmth and dryness favor mummification; moisture and exclusion of air gangrene. This holds good also for the cord when it is separated from the living baby by an additional ligature, and in the dead. Thus, the former forensic axiom that a dry cord proved life, which prevailed for decades after Meckel had demonstrated its fallacy as early as 1853, is absolutely worthless. Thus, fatty substances and moisture of any kind must be avoided as much as possible. Powdered subnitrate of bismuth, or oxide of zinc, or iodoform, or salicylic acid, one part with ten parts of starch, may be dusted round the insertion of the cord and over the stump daily. The latter application is not necessarily useless (from the point of view of antisepsis), for the separation of the cord is a gradual one, and not uniform through the whole thickness of the amnion and the three blood-vessels.

"The size of the sore stump and the rapidity or slowness of cicatrization depend upon the thickness of the cord, the intensity of the line of demarcation, and the reactive inflammation. The latter are most marked in vigorous infants. As a rule the surface is dry a few days after the falling of the cord, and cicatrization complete within twelve or fifteen days after birth. This normal process is, however, disturbed by careless handling, local irritation, and infectious influences. In these cases there is a serous or purulent secretion, and cicatrization may be deferred for many weeks. Under these circumstances local treatment is required. Carbolic acid ought to be avoided, for the newly-born and the infant are easily influenced by its poisonous properties. Solutions of lead, zinc, or alum answer quite well. As before, however, I recommend the powders of zinc oxide, bismuth subnitrate, alum with starch, and salicylic acid with starch or iodoform. Such measures will always prove helpful; to omit them in time of erysipelas or diphtheria is unpardonable. Perchloride of iron, or sulphate of iron, must not be used. Under the hard coagulation formed by its application over the whole wound secretions will accumulate, cannot escape, are absorbed, and produce sepsis. I have seen babies die from applications of iron to the umbilical stump, as I know of women dying for the same reason when the hemorrhages from their uteri or from the lacerated vagina were maltreated in the same manner. —*New York Medical Journal.*

---

### PUERPERAL SEPSIS.

The *Medical and Surgical Reporter* gives a synopsis of a paper by Dr. Lewis S. McMurtry, of Louisville, on "The Indications for Operation in Puerperal Sepsis," as follows: Pre-existing pus-tubes, a uterine fibroid or ovarian dermoid converted by the trauma of labor into activity as an infecting source, should be treated by promp resort to abdominal section. Septic endometritis, with or without putrefactive changes in retained clots and débris, should be relieved by cleansing, antisepsis, and drainage. Thorough intra-uterine drainage and irrigation in appropriate cases arrest the septic process. Curettage in these cases is too extensively used when the septic focus is limited to the uterine cavity, as the granular area of Bumm may be broken through; closed sinuses and veins at the placental site reopened; repeated chills and rising pulse and temperature marking the invasion of new areas of infection. Plugging up the uterine cavity is positively contraindicated; drainage should be facilitated and not obstructed in these cases. Purulent salpingitis, ovarian abscess and suppurative peritonitis, by progressive steps, may extend very rapidly, and the associated peritonitis may be circumscribed or diffuse, consequently careful and deliberate judgment must be exercised before resorting to celiotomy. The time of the operation and the extent to which the operative procedure is to be carried also require sound judgment. Diffuse septic parenchymatous metritis and purulent metritis should be promptly treated by hysterectomy. Puerperal sepsis, wherein the local symptoms are those of diffuse peritonitis without localization of lesions, but where the uterus is presumably the focus of infection, is a grave condition and often justifies exploration and drainage, but hysterectomy will almost invariably prove disastrous.

---

### PYOSALPINX.

Dr. Joseph Price, of Philadelphia, makes an earnest plea in the *Medical and Surgical Reporter* of December 21, 1895, for adherence to the suprapubic operation for removal of pyosalpinx. He condemns the vaginal route of attack of purulent accumulations in the Fallopian tubes because it does not fulfill what is necessary for a perfect or "complete" operation. An operation, to be complete, must remove all that it professes to remove. It must correct all pathological complications and lesions, and leave all surrounding structures in as normal relations as possible. Unrecognized and unrepaired fistulæ, to the number of five or six per cent., following the intra-operation, is alone sufficient reason for its total rejection as one of the most imperfect, inefficient, and unsatisfactory methods ever practiced in gynecology. But, he says, the suprapubic method, as perfectly practiced—free of errors of omission and commission—is the only operation that can give perfect, immediate, and permanent results. The accidents, complications, and sequelæ commonly referred to in discussions of the suprapubic operation—of infection, adhesions, fistula following drainage, and improper ligatures—are all avoidable, except in very feeble patients.

